# The research on the immuno-modulatory defect of Mesenchymal Stem Cell from Chronic Myeloid Leukemia patients

**DOI:** 10.1186/1756-9966-30-47

**Published:** 2011-05-02

**Authors:** Zhu Xishan, An Guangyu, Song Yuguang, Zhang Hongmei

**Affiliations:** 1 Institute of Medical Oncology, Beijing Shijitan Hospital, Capital Medical University, Beijing, 100038, P.R. China

## Abstract

Overwhelming evidence from leukemia research has shown that the clonal population of neoplastic cells exhibits marked heterogeneity with respect to proliferation and differentiation. There are rare stem cells within the leukemic population that possess extensive proliferation and self-renewal capacity not found in the majority of the leukemic cells. These leukemic stem cells are necessary and sufficient to maintain the leukemia. While the hematopoietic stem cell (HSC) origin of CML was first suggested over 30 years ago, recently CML-initiating cells beyond HSCs are also being investigated. We have previously isolated fetal liver kinase-1-positive (Flk1^+^) cells carrying the BCR/ABL fusion gene from the bone marrow of Philadelphia chromosome-positive (Ph^+^) patients with hemangioblast property. Here, we showed that CML patient-derived Flk1^+^CD31^-^CD34^-^MSCs had normal morphology, phenotype and karyotype but appeared impaired in immuno-modulatory function. The capacity of patient Flk1^+^CD31^-^CD34^- ^MSCs to inhibit T lymphocyte activation and proliferation was impaired in vitro. CML patient-derived MSCs have impaired immuno-modulatory functions, suggesting that the dysregulation of hematopoiesis and immune response may originate from MSCs rather than HSCs. MSCs might be a potential target for developing efficacious cures for CML.

## Introduction

Chronic Myeloid Leukemia(CML) is a malignant myeloproliferative disorder originating from a pluripotent stem cell that expresses the BCR/ABL oncogene and is characterized by abnormal release of the expanded, malignant stem cell clone from the bone marrow into the circulation[1,2]. The discovery of the Philadelphia chromosome followed by identification of its BCR/ABL fusion gene product and the resultant constitutively active P210 BCR/ABL tyrosine kinase prompted the unravelling of the molecular pathogenesis of CML. However, regardless of greatly reduced mortality rates with BCR/ABL targeted therapy, most patients harbor quiescent CML stem cells that may be a reservoir for disease progression to blast crisis. Under steady-state conditions, these cancer stem cells are localized in a microenvironment known as the stem cell "niche", where they are maintained in an undifferentiated and quiescent state. These niches are critical for regulating the self-renewal and cell fate decisions, yet why and how these cells are recruited to affect leukemia progression are not well known.

Local secretion of proteases has been implicated in this tumor-stroma crosstalk. Matrix metalloproteinase-9 (MMP-9) is one of the proteases that has the preferential ability to degrade denatured collagens (gelatin) and collagen type IV, the 2 main components of basement membranes and therefore plays a critical role in tumor progression and metastasis[3,4]. Previous studies have demonstrated localization of MMP-9 on the plasma membrane of various tumor cells[5-7] and recently, the role of MMP-9 in CML pathogenesis has became a focus of attention[8-11]. But the research is mainly focusing on the MMP-9 inducing molecules[12-14] or the effect of MMP-9 inhibitors[15]. However, it has become clear that the role of MMP-9 in CML is not limited to simple extracellular matrix (ECM) degradation[16]. The regulation of MMP-9 is found to be involved in multiple pathways induced by different kinds of cytokines in different cell types and illness[17,18]. Therefore, it is necessary to verify a specific MMP-9 induced pathway in a given cell type.

Recent research[6,10,4] showed that T lymphocytes isolated from CML patients suppressed the forming of CFU-GM (colony forming unit-granulocyte and macrophage) and CFU-E (colony forming unit-erythroid) and furthermore this kind of inhibition could be blocked by CsA(cyclosporine A)[19,20];besides, the rate of the forming of the HSCs (hematopoietic stem cells) increased with the removal of T lymphocytes. Therefore, immunological inhibitors like CsA. and ATG (anti-human thymocyte globulin) was helpful for CML patients and was widely used in clinic therapy[21-23]. All these evidence indicated there might existed immunological abnormalities, that is, the T lymphocytes in CML might existed in a unusually activated state leading to self injury.

Besides HSCs, there also existed another kind of stem cells called MSCs (Mesenchymal Stem Cells), they could differentiated into stroma cells and acted as the "niche" in the micro-environment[24]. MSCs also had the immunological regulation ability and were believed to be the "immune protection site" in the cells environment. So, we believed that MSCs might play important role in the pathogenesis of CML, but there was no article examined the immunological function of MSCs.

Previous studies[19,21] from our laboratory have identified Flk1^+ (^fetal liver kinase-1 positive) CD31^-^CD34^- ^cells carrying the BCR/ABL fusion gene from the bone marrow of Philadelphia chromosome positive (Ph^+^) patients with CML and found that these cells could differentiate into malignant blood cells and phenotypically defined endothelial cells at the single-cell level, suggesting these cells have the properties of hemangioblasts. The main purpose of our article was to examine the immune characteristics of Flk1^+^CD31^-^CD34^- ^MSC in CML and analyse if there existed abnormalities comparing with the healthy donors.

## Patients, materials, and methods

### Patient samples

20 patients with newly diagnosed CML (12 male and 8 female, aged 17-63 years) were recruited in this study(table 1). All were Ph^+ ^patients with CML in chronic phase as revealed by bone marrow histology and cytogenetic analysis. The immunophenotypes of thawed cells were quite variable. None was treated with hydroxyurea or interferon before. The control samples were from 20 healthy donors (12 male and 8 female, aged 21-60 years). Bone marrow samples were collected after obtaining informed consent according to procedures approved by the Ethics Committee at the 309^th ^Hospital of Peoples Liberation Army.

**Table 1:** The general conditions of the patients

Patient	Age	Sex	Diagonosis	Diagnosis time	Ph chromosome	Immunosuppressive therapy
1	84	F	CML	Aug-04	positive	yes
2	54	M	CML	Jun-87	positive	yes
3	56	M	CML	May-99	positive	yes
4	49	M	CML	Feb-87	positive	yes
5	66	M	CML	Aug-04	positive	yes
6	40	F	CML	Feb-05	positive	No
7	50	F	CML	Sep-04	positive	No
8	76	F	CML	Aug-04	positive	No
9	64	F	CML	Dec-05	positive	No
10	55	M	CML	Apr-00	positive	yes
11	49	M	CML	Feb-05	positive	No
12	51	M	CML	Jun-01	positive	yes
13	40	F	CML	Dec-05	positive	No
14	43	F	CML	Dec-05	positive	No
15	60	M	CML	Nov-05	positive	No

M: male; F:female;

### Cell preparations and culture

Isolation and culture of bone marrow-derived CML hemangioblasts were performed as described previously with some modifications[19,21]. Briefly, mononuclear cells were separated by a Ficoll-Paque gradient centrifugation (specific gravity 1.077 g/mL; Nycomed Pharma AS, Oslo, Norway) and the sorted cells were plated at concentration of 1 cell/well by limiting dilution in a total of 96 × 10 wells coated with fibronectin (Sigma, St Louis, MO) and collagen (Sigma) for each patient. Culture medium was Dulbecco modified Eagle medium and Ham F12 medium (DF12) containing 40% MCDB-201 medium complete with trace elements (MCDB) (Sigma), 2% fetal calf serum (FCS; Gibco Life Technologies, Paisley, United Kingdom), 1 × insulin transferrin selenium (Gibco Life Technologies), 10^-9 ^M dexamethasone (Sigma), 10^-4 ^M ascorbic acid 2-phosphate (Sigma), 20 ng/mL interleukin-6 (Sigma), 10 ng/mL epidermal growth factor (Sigma), 10 ng/mL platelet-derived growth factor BB (Sigma), 50 ng/mL fetal liver tyrosine kinase 3 (Flt-3) ligand (Sigma), 30 ng/mL bone morphogenetic protein-4 (Sigma), 100 U/mL penicillin and 100 ug/mL streptomycin (Gibco Life Technologies) at 37°C and a 5% CO_2 _humidified atmosphere. Culture media were changed every 4 to 6 days.

### FISH analysis

We cultured BCR/ABL^+ ^hemangioblasts from male CML patients (n = 12) and Y chromosome was detected using a probe (CEP Y Spectrum Red; Vysis, Downers Grove, IL) according to the manufacturer's instructions. Normal cells showed 2 red abl signals and 2 green bcr signals. BCR/ABL^+ ^hemangioblasts showed a single red and a single green signal representing normal abl and bcr genes and the yellow signal representing fusion of abl and bcr genes.

### Fluorescence activated cell sorting (FACS)

For immunophenotype analysis, expanded clonal cells were stained with antibodies against Flk1, CD29, CD31, CD34, CD44, CD45, CD105, (all from Becton Dickinson Immunocytometry Systems, Mountain View, CA). For intracellular antigen detection, cells were first fixed in 2% paraformaldehyde (Sigma) for 15 minutes at 4°C and permeabilized with 0.1% saponin (Sigma) for 1 hour at room temperature. Cells were washed and labeled with fluorescein isothiocyanate (FITC) conjugated secondary goat antimouse, goat antirabbit, or sheep antigoat antibodies (Sigma), then washed and analyzed using a FACS Calibur flow cytometer (Becton Dickinson, San Jose, CA).

### Mitogen proliferative assays

Inmitogen proliferative assays, triplicate wells containing responder 1 × 105 MNCs were cultured with 50 g/ml PHA (Roche, USA) in a total volume of 0.1 ml medium at 37°C in 5% CO2, and Flk1+CD31-CD34- MSCs were added on day 0. Irradiated Flk1+CD31-CD34- MSCs (30 Gy) were cocultured with the MNCs at different ratios (MSCs to MNCs = 1:2, 1:10, 1:100). Control wells contained only MNCs. Cultures were pulsed with 1 Ci/well [3H]-TdR (Shanghai Nucleus Research Institute, China) on day 2, and harvested 18 h laterwith a Tomtec (Wallac Inc., Gaithersburg, MD) automated harvester. Thymidine uptake was quantified using a liquid scintillation and luminescence counter (Wallac TRILUX).

### Mixed lymphocyte reaction assays (MLR)

Blood mononuclear cells (MNCs) were prepared from normal volunteers' peripheral blood by Ficoll-Paque density gradient centrifugation and suspended inRPMI 1640 medium supplemented with 10% (vol/vol) FCS, 2 mM l-glutamine,0.1 mM nonessential amino acids (Life Technologies, Grand Island, NY), 1 mM sodium pyruvate, 100 U/mL penicillin,

### Effect of MSCs on T cell cycle

MSCs and MNCs were prepared as described before. T cells, stimulated with PHA (50 g/ml, final concentration) stimulation for 3 days, were cultured alone or cocultured with MSCs (derived from normal and MDS patient) or 3T3 cell line, then harvested and quantified. One million T cells were fixed with 70% cold ethanol at 4°C for 30 min, washed with PBS twice, and stained with 50 g/ml PI (Sigma, USA) at room temperature for 5 min. Data were analyzed with Mod-FIT software.

### Effect of MSCs on T cell activation

MSCs and MNCs were prepared as described before, respectively. T cells were cultured alone or cocultured with prepared MSCs and stimulated with PHA (50 g/ml final concentration). The expression of CD25 (BD, USA) and CD69 (BD, USA) was detected by flow cytometry at 24 h, and CD44 (BD, USA) was detected at 72 h.

### Effect of MSCs on T cell apoptosis

MSCs and MNCs were prepared as described before. T cells were cultured alone or cocultured withMSCs with PHA (50 g/ml final concentration) stimulation for 3 days, then harvested and quantified, stained with Annexin-V kit (BD, USA), and analyzed by flow cytometry (FACS Vantage).

### RNA-i experiments

The si-RNA sequence targeting human MMP-9 (from mRNA sequence; Invitrogen online) corresponds to the coding region 377-403 relative to the first nucleotide of the start codon (target = 5'-AAC ATC ACC TAT TGG ATC CAA ACT AC-3'). Computer analysis using the software developed by Ambion Inc. confirmed this sequence to be a good target. si-RNAs were 21 nucleotides long with symmetric 2-nucleotide 3'overhangs composed of 2'-deoxythymidine to enhance nuclease resistance. The si-RNAs were synthesized chemically and high pressure liquid chromatography purified (Genset, Paris, France). Sense si-RNA sequence was 5'-CAU CAC CUA UUG GAU CCA AdT dT-3'. Antisense si-RNA was 5'-UUG GAU CCA AUA GGU GAU GdT dT-3'. For annealing of si-RNAs, mixture of complementary single stranded RNAs (at equimolar concentration) was incubated in annealing buffer (20 mM Tris-HCl pH 7.5, 50 mM NaCl, and 10 mM MgCl_2_) for 2 minutes at 95°C followed by a slow cooling to room temperature (at least 25°C) and then proceeded to storage temperature of 4°C. Before transfection, cells cultured at 50% confluence in 6-well plates (10 cm^2^) were washed two times with OPTIMEM 1 (Invitrogen) without FCS and incubated in 1.5 ml of this medium without FCS for 1 hour. Then, cells were transfected with MMP-9-RNA duplex formulated into Mirus *Trans*IT-TKO transfection reagent (Mirus Corp, Interchim, France) according to the manufacturer's instructions. Unless otherwise described, transfection used 20 nM RNA duplex in 0.5 ml of transfection medium OPTIMEM 1 without FCS per 5 × 10^5 ^cells for 6 hours and then the medium volume was adjusted to 1.5 ml per well with RPMI 2% FCS. SilencerTM negative control 1 si-RNA (Ambion Inc.) was used as negative control under similar conditions (20 nM). The efficiency of silencing is 80% in our assay.

### Enzyme-linked Immunoadsorbent Assays

This was carried out according to the manufacturer's recommendations (Oncogene Research Products). Results were compared with those obtained with serially diluted solutions of commercially purified controls. Anti-human cytokine antibodies (R&D Systems, Minneapolis, MN) was added at 0.4 ug/ml in 0.05 M bicarbonate buffer (pH 9.3) to 96-well, U-bottom, polyvinyl microplates (Becton Dickinson and Co., Oxnard, CA) and the cell number was 1 × 10^5^/100 ul. After incubation overnight at 4°C, the plates were washed and blocked with 1% gelatin for 1 hour. Samples (50 ul) or standard protein diluted in 0.5% gelatin were added to the wells. After incubation for 1 hour at 37°C, the plates were washed again, and 50 ng/ml biotinylated antimouse antibody (R&D Systems) was added for 1 hour at 37°C. The plates were then washed and incubated with streptavidin-HRP for 1 hour at 37°C. After washing, 0.2 mM ABTS (Sigma Chemical Co.) was added to the wells, and after 10 minutes, the colorimetric reaction was measured at 405 nm with an ELISA reader VERSAmax (Molecular Devices, Sunnyvale, CA).

### Western blot

CML hemangioblasts were harvested at specific times after treatment with regents as indicated in each experiment. Cells were mixed with loading buffer and subject to electrophoresis. After electrophoresis, proteins were transferred to polyvinyl difluoride membranes (Pall Filtron) using a semidry blotting apparatus (Pharmacia) and probed with mouse mAbs, followed by incubation with peroxidase-labeled secondary antibodies. Detection was performed by the use of a chemiluminescence system (Amersham) according to the manufacturer's instructions. Then membrane was striped with elution buffer and reprobed with antibodies against the nonphosphorylated protein as a measure of loading control. Controls for the immnoprecipitation used the same procedure, except agarose beads contained only mouse IgG.

### Statistics

Statistical analysis was performed with the statistical SPSS 13.0 software. The paired-sample t-testwas used to test the probability of significant differences between samples. Statistical significance was defined as p < 0.05.

## Results

### The biological characteristics of CML hemangioblasts

**Figure 1:**
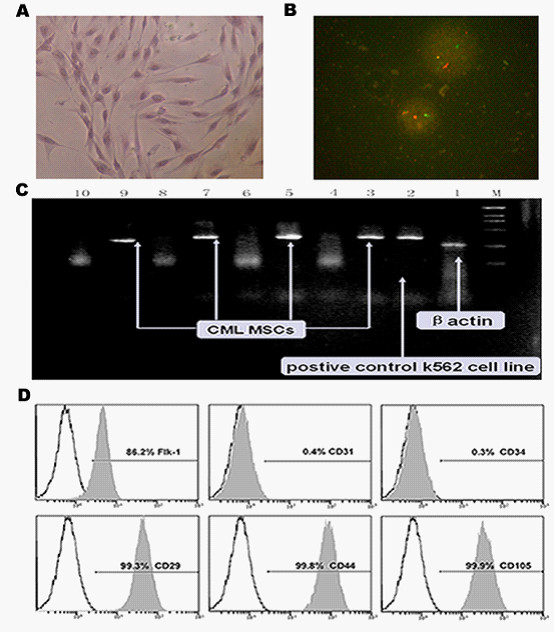
**Biological characteristics of the CML MSCs**. **(A) **The morphology of hemangioblasts from CML (Magnification × 200). **(B) **BCR/ABL fusion gene was detected by FISH (yellow signal is the positive one) in CML hemangioblasts from male patients. **(C) **BCR/ABL fusion gene was detected by RT-PCR(line4,6,8,10 correspond to non-special amplification).**(D) **Isotype analysis showed they were all persistently negative for CD34 and CD31 but positive for Flk1, CD29, CD44 and CD105.

To establish the characteristics of CML hemangioblasts, we first examined the morphology, phenotype and growth patterns of them respectively. Results showed that they persistently displayed fibroblast-like morphology (Figure 1A) and CML specific BCR/ABL oncogene was observed by FISH analysis (Figure 1B) and PCR (Figure 1C) in CML hemangioblasts. Isotype analysis indicated they were all persistently negative for CD34 and CD31 but positive for Flk1, CD29, CD44 and CD105 (Figure 1D).

### Immunomodulatory decrease on T cell proliferation

**Figure 2:**
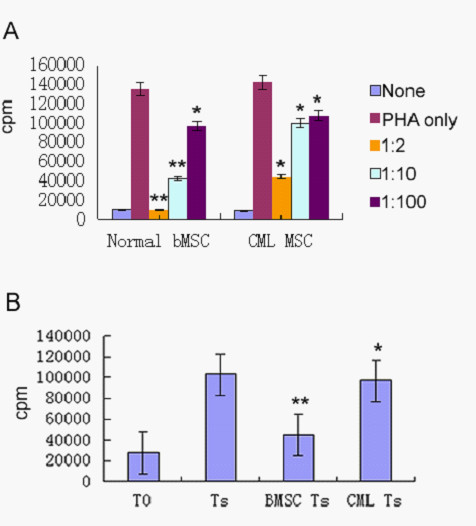
**The effects of Flk-1+CD31-CD34- MSCs on T lymphocyte proliferation**. (A) The effects of Flk-1+CD31-CD34- MSCs on T lymphocyte proliferation in mitogen proliferative assays. There are three groups, including nonstimulated T cells (none), PHA-stimulated T cells (Ts) and PHA-stimulated T cells cocultured with MSC at different ratios (MSC to T cell = 1:2, 1:10, :100). Data are shown as means ± S.D. of three independent experiments (*p < 0.05,**p < 0.005 vs. Ts). (B) The effects of Flk-1+CD31-CD34- MSCs on T lymphocyte proliferation in MLR. Flk-1+CD31-CD34- MSCs at 1:10 ratios (irradiated MSCs to T cells); there are four groups, including nonstimulated responder T cells (T0), irradiated stimulator cells plus responder T cells; normalMSC plusMLR (BMSC Ts), CML-derived MSC plus MLR (CML Ts). Data are shown as means ± S.D. of three independent experiments (*p ≥ 0.05,**p = 0.001 vs. Ts)

To analyse immunomodulatory effects on T cell proliferation, irradiated MSCs were added to mitogen-stimulated T cell proliferation reactions and mixed lymphocyte reactions (MLR). A previous study showed that MSCs from healthy volunteers could obviously inhibit the proliferation of T cells not only stimulated with mitogen but also in MLR. Additionally, this inhibitory effect occurred in a dose-dependent manner. In mitogen-stimulated T cell proliferation assays, the proliferation of T cells at 1:2 ratio (MSCs to MNCs) was significantly inhibited to about 1% with normal MSCs, but proliferation at the same ratiowas inhibited only to about 37% with CML-derived MSCs (compared with co-culture system of normal MSCs, p < 0.05). Similarly, inhibitory rates were impaired at 1:10 ratio (MSCs to MNCs) in CML-derived MSCs (compared with co-culture system of normal MSCs, p < 0.05). Also the inhibitory effect was dose dependent in CML-derived MSCs. (Figure 2A). In MLR, a similar impaired inhibitory effect with MDS-derived MSCs was observed. (Figure 2B)

### Immunomodulatory attenuation of MSCs on T cell cycle

**Figure 3:**
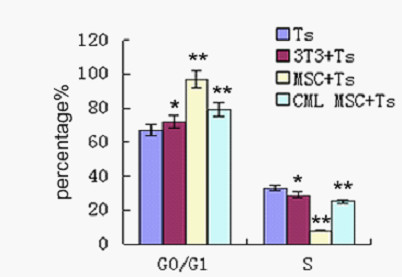
**Effects ofMSCs on T cell cycle**. Flk-1+CD31-CD34- MSCs or 3T3 at 1:10 ratios (MSCs to T cells); the data are expressed as mean ± S.D. Of triplicates of five separate experiments with similar results. Cell cycles of PHA-stimulated T cells were analyzed in T cells alone (Ts), cocultured with MSCs (MSC + Ts) group andMSCs derived from CML patient group (CML MSC + Ts). 3T3 cell line was used as control (3T3 + Ts). Data are shown as means ± S.D. of five independent experiments (*p ≥ 0.05, **p < 0.05 vs. Ts)

A previous study showed that MSCs could silence T cells in G0/G1 phase, which might be one of the possible mechanisms of MSC's inhibitory effect on T cells. When the inhibitory effect of CML-derived MSC on T cell proliferation was impaired, the related inhibitory effect on cell cycle was analyzed. In a PHA-stimulating system without MSC co-culture, there were 67.3 ± 3.7% and 28.4 ± 2.9% T cells in G0/G1 phase and S phase, respectively. When normal MSCs were present in co-culture, the percentages of T cells in G0/G1 phase and S phase were 94.0 ± 1.9% and 3.1 ± 1.9%, respectively (compared with PHA stimulated T cells, p < 0.05). MSCs from healthy volunteers could have most of their T cells in G0/G1 phase with fewer cells entering S phase. However, T cells in G0/G1 phase and S phase remained 74.5 ± 1.2% and 22.1 ± 2.4% in the co-culture system of CML-derived MSCs (compared with co-culture system of normal MSCs, p < 0.05). This result was confirmed by five independent tests (Figure 3). The 3T3 cell line was used as a control, and no effects on cell cycle were observed (70.3 ± 3.1% in G0/G1 and 27.3 ± 5.1% in S, respectively (compared with PHA stimulated T cells, p > 0.05). These results suggested that the inhibitory effect of CML-derived MSCs on cell cycle arrest was also impaired.

### Impaired effects of MSCs on T cell activation

**Figure 4:**
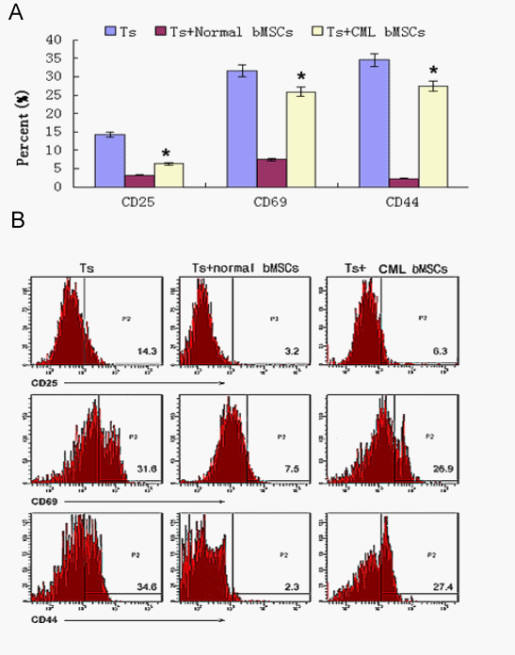
**Effects of Flk-1+CD31-CD34- MSCs on T lymphocyte activation**. Flk-1+CD31-CD34- MSCs at 1:10 ratios (MSCs to T cells); the data are expressed as mean ± S.D. of triplicates of five separate experiments with similar results. Activators of T cells were analyzed including CD25, CD69, and CD44. The activation of T cells was analyzed in T cells alone (Ts), normal MSC cocultured with activated T cells (BMSC + Ts), and CML-derived MSC cocultured with activated T cells (MDS MSC + Ts). Data are shown as means ± S.D. of five independent experiments (*p ≥ 0.05,**p < 0.05 vs. Ts)

MSCs from CML patients could significantly inhibit activation of T cells. The percentage of CD25, CD69 and CD44 in PHA induced T lymphocyte was 12.3 ± 3.5%, 34.5 ± 5.9% and 29.4 ± 7.0% respectively. But they were 3.1 ± 2.3%, 6.4 ± 3.2% and 2.1 ± 1.7% when co-cultured with normal hemangioblasts and, when co-cultured with CML hemangioblasts, they were 5.4 ± 2.3%, 31.5 ± 6.8% and 24.5 ± 3.6% respectively. All data presented here were confirmed by repeated tests (Figure 4). These results also indicated that MSCs from CML patients were impaired in their immuno-modulatory function.

### Dampening effect of MSCs on T cell apoptosis

**Figure 5:**
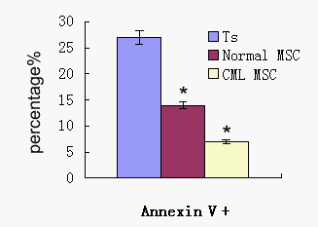
**Effect of MSCs on T cell apoptosis**. Flk-1+CD31-CD34- MSCs at 1:10 ratios (MSCs to T cells); the data are expressed as mean ± S.D. of triplicates of five separate experiments with similar results. The test was conducted by Annexin-V and PI double staining and analyzed by flow cytometry. Apoptosis of T cells was analyzed in T cells alone (Ts), normalMSC cocultured with activated T cells (MSC + Ts), and CML patient-derived MSC cocultured with activatedT cells (CMLMSC + Ts). Annexin V+means the cells were PI negative and Annexin V positive. Data are shown as means ± S.D. of five independent experiments (*p < 0.05 vs. Ts)

In apoptosis tests, we have observed that MSCs from healthy volunteers could significantly dampen the effect of activation-induced apoptosis of T cells. Following stimulation with PHA for 3 days, the rate of apoptosis of T cells was 23.37 ± 2.71%. When PHA-stimulated T cells were cocultured with MSCs obtained from healthy volunteers, the percentage of apoptotic T cells decreased to 14.1 ± 0.65% (compared with PHA stimulated T cells, p < 0.05). In the same condition, the apoptosis percentage of T cells co-cultured with MDS-derived MSCs further decreased to 8.36 ± 1.31% (compared with co-culture systemof normalMSCs, p < 0.05). We repeated the experiment five times to confirm this result (Figure 5). These results suggested the dampening effect of CML-derived MSCs on activation-induced T apoptosis seemed to be enhanced.

### Efficient extinction of MMP-9 expression in HT1080 cells by RNAi strategy and the concomitantly upregulation of s-ICAM-1

**Figure 6:**
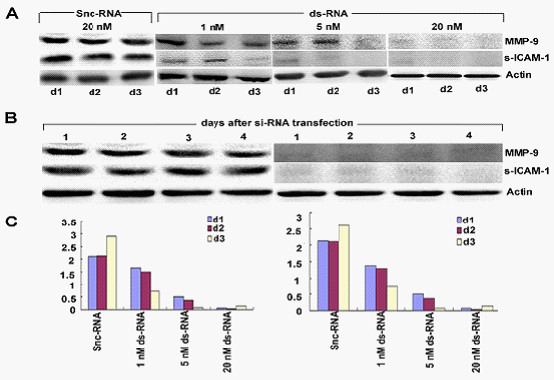
**Efficient inhibition of MMP-9 in CML MSC using RNAi**. (A) The cDNAs from snc-RNA (20 nM) and ds-RNA (1-20 nM) cells cultured for up 3 days were used as templates for PCR reactions using specific primers for MMP-9 and ICAM-1. (B) The cDNAs from snc-RNA (20 nM) and ds-RNA (20 nM) cells cultured for up 4 days were used as templates for PCR reactions using specific primers for MMP-9 or 18 S ribosomal RNA. (C) MMP-9 and s-ICAM-1 production (ng/ml) in the culture supernatants of CML snc-RNA (20 nM) or ds-RNA (1-20 nM) cells were determined by enzymelinked immunosorbent assays.

We used an RNAi method to target MMP-9 in the CML MSC and the constructs we designed encoded an RNA that targets the MMP-9 mRNA. The target sequence had no homology with other members of the MMP family. The ds-RNA and Silencer negative control si-RNA (snc) were each tested for their ability to suppress MMP-9 specifically. We first assessed whether RNAi was dose and time-dependent. A MMP-9 dependent ds-RNA-mediated inhibition was observed in a dose and time dependent manner (Figure 6A). The time-course assay performed with 20 nM ds-RNA-transfected CML MSC showed that the induced MMP-9 silencing could be maintained for at least 3 days (Figure 6B). Besides, serum ICAM-1 was concomitantly changing with MMP-9. The Western blotting results were confirmed by enzyme-linked immunoadsorbent assay. CML snc-RNA-transfected cells cultured up to 3 days spontaneously released high amount of MMP-9 into the culture conditioned medium whereas ds-RNA-transfected cells showed a marked time- and dose- dependent inhibition in MMP-9 protein levels. Importantly, levels of s-ICAM-1 were also affected with ds-RNA transfection (Figure 6C).

## Discussion

MSC isolated from different tissues had immune regulation ability not only in vivo but in vitro and it might consist the "immune protection site" in human body[25,26]. Considering their richness in source, availability for expansion, and most importantly, their robust immuno-modulatory activity, MSCs appear to be a primary candidate for cellular therapy in immune disorders[12,16,27]. In normal physiological conditions, MSCs are very scarce (one MSC per 10,000-100,000MNC), therefore, normal immune responses against foreign antigens are not affected. This is consistent with in vitro results showing that immuno-suppressive function was abolished when the ratio of MSC to T cells was less than 1:100. However, once a large number of MSCs were infused for immune therapy, influx of MSC in the circulation and bone marrow could bring the hypersensitive immune response to normal. Moreover, MSC infusion could not only modulate immune responses but enhance the hematopoietic microenvironment. Transplantation of MSCs offers bright prospects in developing new therapies for blood diseases caused by an abnormal immune system and impaired hematopoietic microenvironment. To date, MSCs have been used to treat GVHD, which is a disorder of hyper-immunoresponse, and shown to be effective clinically[28,29].

Chronic myeloid leukemia is a clonal hematopoietic stem cell disorder characterized by the t(9;22) chromosome translocation and resultant production of the constitutively activated *BCR/ABL *tyrosine kinase[30]. Interestingly, this BCR/ABL fusion gene, was also detected in the endothelial cells of patients with CML, suggesting that CML might originate from hemangioblastic progenitor cells that can give rise to both blood cells and endothelial cells. Although Interferon-α, Intimab(a *BCR/ABL *tyrosine kinase inhibitor) and stem cell transplantations are the standard therapeutic options, transplant-related morbidity from graft-versus-host disease and mortality rates of 10% to 20% have greatly reduced the allogeneic hematopoietic cell transplantation in clinics[31], while interferon-α is only effective in some patients to some degree and chemotherapeutic intervention does not result in prolonged overall survival[32,33] and the reason is possibly due to some unknown biology of the CML immune regulation[34].

We conducted this study of CML patient-derived MSCs to evaluate the safety and effectiveness of autologous MSCs in treating CML. We tested the karyotype and genetic changes of in vitro-expanded MSCs for safety evaluation. The immuno-modulatory function of MSCs was also examined. The investigation of CML patient-derived MSCs could help to further elucidate etiology and pathology of CML. Specifically, the answers to questions of whether gene aberrations exist in MSCs and whether the functions of MSCs are impaired are crucial for understanding of CML development and finding effective treatments.

We utilised Flk1+CD31-CD34- MSCs from CML patients for 4-6 passages, and there were chromosomal abnormities, indicating that mutation of CML happened at the hematoangioblast level[35]. We thereby hypothesized that malignant mutation existed in stem cells more primordial than HSCs. Data from functional tests proved that CML-derived MSCs had abnormal immuno-modulatory function, although their MSCs showed normal karyotype. An inhibitory effect on T cell proliferation is an important characteristic of MSC in immuno-modulatory action. A previous study, in accordance with another report, suggested that the inhibitory effect on T cell proliferation might be through cell cycle arrest. MSCs from healthy volunteers could obviously block T cells in G0/G1 phase. In this study, inhibitory effects of MDS-derived MSCs on T cell proliferation were obviously impaired. Moreover, no significant cell cycle arrest was observed in PHA-stimulated T cells cocultured with CML-derived MSCs. In addition, an inhibitory effect on T cell activation is another key point of immuno-modulatory function for MSCs, although there are still disputes[21,22]. CD25, CD69 and CD44 are candidates for T cell activation in different phases. In our study, MSCs from healthy volunteers showed significant inhibitory effects on expression of T cell activation markers, but MSCs from CML patients showed very limited inhibitory effects. These results suggested that CML-derived MSCs have immunologic abnormalities and their application in immuno-modulation might be limited.

Normally, the invasion and metastasis by malignant tumor cells consists of three major steps: the receptor-mediated adhesion of tumor cells to the extracellular matrix, the degradation of the extracellular matrix by the proteinase secreted by the tumor cells, and the transfer and proliferation of tumor cells[36]. So, the loose of ECM and secreted cytokines are important for the metastasis of the tumor cells from the primary tumor[37]. Pathological conditions will change the tumor cell fate leading to invasion and metastasis[38], Local secretion of proteases have been implicated in this tumor-stroma crosstalk. Matrix Metalloproteinase-9 (MMP-9) is one of them which has the preferential ability to degrade denatured collagens (gelatin) and collagen type IV, the 2 main components of basement membranes and therefore plays a critical role in tumour progression and metastaisis[39]. Moreover, its expression increases with the increased or greater proliferation of tumor cells.

We used a ds-RNA to interfere with the expression of MMP-9 gene in CML MSC and our findings support the conclusion that MMP-9 constitutes a trigger for the switch between adhesive and invasive states in CML MSC by changing the ICAM-1 from membrane-anchored state to solvable one leading to tumor cell immune evasion and metastasis.

In conclusion, the immune function of CML patient-derived MSCs showed that their immuno-modulatory ability, compared to MSCs from healthy volunteers, was impaired, whichmight be a cause for an abnormal hematopoietic environment. This indicates that autologous MSCs transplantation might be futile. Instead, allogenic MSCs transplantation might be a better choice to ameliorate CML.

## Competing interests

The authors declare that they have no competing interests.

## Authors' contributions

ZH carried out the molecular genetic studies, participated in the sequence alignment and drafted the manuscript. AG carried out the immunoassays. SY participated in the design of the study and performed the statistical analysis. All authors read and approved the final manuscript.

## References

[B1] Barnes DJ Melo JV Primitive, quiescent and difficult to kill: the role of non-proliferating stem cells in chronic myeloid leukemia Cell Cycle 2006 5 2862 2866 10.4161/cc.5.24.3573 17172863

[B2] Jørgensen HG Allan EK Jordanides NE Mountford JC Holyoake TL Nilotinib exerts equipotent antiproliferative effects to Imatinib and does not induce apoptosis in CD34+CML cells Blood 2007 109 4016 4019 10.1182/blood-2006-11-057521 17213283

[B3] Jørgensen HG Copland M Allan EK Jiang X Eaves A Eaves C Holyoake TL Intermittent exposure of primitive quiescent chronic myeloid leukemia cells to granulocyte-colony stimulating factor in vitro promotes their elimination by Imatinib mesylate Clin Cancer Res 2006 12 626 633 10.1158/1078-0432.CCR-05-0429 16428509

[B4] Ries C Pitsch T Mentele R Zahler S Egea V Nagase H Jochum M Identification of a novel 82 kDa proMMP-9 species associated with the surface of leukaemic cells: (auto-)catalytic activation and resistance to inhibition by TIMP-1 Biochem J 2007 405 3 547 58 10.1042/BJ20070191 17489740 PMC2267301

[B5] Yu Q Stamenkovic I Cell surface-localized matrix metalloproteinase-9 proteolytically activates TGF-β and promotes tumor invasion and angiogenesis Genes Dev 2000 14 163 176 10652271 PMC316345

[B6] Fridman R Toth M Chvyrkova I Meroueh S Mobashery S Cell surface association of matrix metalloproteinase-9 (gelatinase B) Cancer Metastasis Rev 2003 22 153 166 10.1023/A:1023091214123 12784994

[B7] Stefanidakis M Koivunen E Cell-surface association between matrix metalloproteinases and integrins: role of the complexes in leukocyte migration and cancer progression Blood 2006 108 1441 1450 10.1182/blood-2006-02-005363 16609063

[B8] Baran Y Ural AU Gunduz U Mechanisms of cellular resistance to imatinib in human chronic myeloid leukemia cells Hematology 2007 12 6 497 503 10.1080/10245330701384179 17852433

[B9] Kim JG Sohn SK Kim DH Baek JH Lee NY Suh JS Clinical implications of angiogenic factors in patients with acute or chronic leukemia: hepatocyte growth factor levels have prognostic impact, especially in patients with acute myeloid leukemia Leuk Lymphoma 2005 46 6 885 91 10.1080/10428190500054491 16019534

[B10] Kaneta Y Kagami Y Tsunoda T Ohno R Nakamura Y Katagiri T Genome-wide analysis of gene-expression profiles in chronic myeloid leukemia cells using a cDNA microarray Int J Oncol 2003 23 3 681 91 12888904

[B11] Bruchova H Borovanova T Klamova H Brdicka R Gene expression profiling in chronic myeloid leukemia patients treated with hydroxyurea Leuk Lymphoma 2002 43 6 1289 95 10.1080/10428190290026358 12152998

[B12] Janowska-Wieczorek A Majka M Marquez-Curtis L Wertheim JA Turner AR Ratajczak MZ Bcr-abl-positive cells secrete angiogenic factors including matrix metalloproteinases and stimulate angiogenesis in vivo in Matrigel implants Leukemia 2002 16 6 1160 6 10.1038/sj.leu.2402486 12040448

[B13] Narla RK Dong Y Klis D Uckun FM Bis(4,7-dimethyl-1, 10-phenanthroline) sulfatooxovanadium(I.V.) as a novel antileukemic agent with matrix metalloproteinase inhibitory activity Clin Cancer Res 2001 7 4 1094 101 11309362

[B14] Sun X Li Y Yu W Wang B Tao Y Dai Z MT1-MMP as a downstream target of BCR-ABL/ABL interactor 1 signaling: polarized distribution and involvement in BCR-ABL-stimulated leukemic cell migration Leukemia 2008 22 5 1053 6 10.1038/sj.leu.2404990 17943163 PMC2430881

[B15] Ries C Loher F Zang C Ismair MG Petrides PE Matrix metalloproteinase production by bone marrow mononuclear cells from normal individuals and patients with acute and chronic myeloid leukemia or myelodysplastic syndromes Clin Cancer Res 1999 5 5 1115 24 10353746

[B16] Kaneta Y Kagami Y Tsunoda T Ohno R Nakamura Y Katagiri T Genome-wide analysis of gene-expression profiles in chronic myeloid leukemia cells using a cDNA microarray Int J Oncol 2003 23 3 681 91 12888904

[B17] Sang-Oh Yoon Sejeong Shin Ho-Jae Lee Isoginkgetin inhibits tumor cell invasion by regulating phosphatidylinosito 3 kinase/Akt dependent matrix metalloproteinase-9 expression Mol Cancer Ther 2006 5 11 344 349 10.1158/1535-7163.MCT-06-0321 17121913

[B18] Anand P Sundaram C Jhurani S Kunnumakkara AB Aggarwal BB Curcumin and cancer: an "old-age" disease with an "age-old" solution Cancer Lett 2008 267 1 133 64 10.1016/j.canlet.2008.03.025 18462866

[B19] Fang Baijun Zheng Chunmei Liao Lianming Shi Mingxia Yang Shaoguang Zhao RCH Identification of Human Chronic Myelogenous Leukemia Progenitor Cells with Hemangioblastic Characteristics Blood 2005 105 7 2733 40 10.1182/blood-2004-07-2514 15591120

[B20] Reyes M Lund T Lenvik T Aguiar D Koodie L Verfaillie CM Purification and ex vivo expansion of postnatal human marrow mesodermal progenitor cells Blood 2001 98 2615 25 10.1182/blood.V98.9.2615 11675329

[B21] Guo H Fang B Zhao RC Hemangioblastic characteristics of fetal bone marrow-derived Flk1(+)CD31(-)CD34(-) cells Exp Hematol 2003 31 650 613 10.1016/S0301-472X(03)00087-0 12842710

[B22] Yunbiao Lu Larry M Wahl. Production of matrix metalloproteinase-9 by activated human monocytes involves a phosphatidylinositol-3 kinase/Akt/IKK/NF-κB pathway J Leuk Bio 2005 78 259 65 10.1189/jlb.0904498 15800029

[B23] Gustin JA Ozes ON Akca H Pincheira R Mayo LD Li Q Guzman JR Korgaonkar CK Donner DB Cell type-specific expression of the IκB kinases determines the significance of phosphati-dylinositol 3-kinase/Akt signaling to NF-κB activation J Biol Chem 2004 279 1615 1620 14585846 10.1074/jbc.M306976200

[B24] Palamà IE Leporatti S de Luca E Di Renzo N Maffia M Gambacorti-Passerini C Rinaldi R Gigli G Cingolani R Coluccia AM Imatinib-loaded polyelectrolyte microcapsules for sustained targeting of BCR-ABL+ leukemia stem cells Nanomedicine (Lond) 2010 5 3 419 31 10.2217/nnm.10.8 20394535

[B25] Karanes C Nelson GO Chitphakdithai P Agura E Ballen KK Bolan CD Porter DL Uberti JP King RJ Confer DL Twenty years of unrelated donor hematopoietic cell transplantation for adult recipients facilitated by the National Marrow Donor Program Biol Blood Marrow Transplant 2008 14 9 Suppl 8 15 9 18721775 10.1016/j.bbmt.2008.06.006

[B26] Martin MG Dipersio JF Uy GL Management of the advanced phases of chronic myelogenous leukemia in the era of tyrosine kinase inhibitors Leuk Lymphoma 2008 29 1 10 10.1080/10428190802517765 19117213

[B27] Martinelli G Soverini S Iacobucci I Baccarani M Intermittent targeting as a tool to minimize toxicity of tyrosine kinase inhibitor therapy Nat Clin Pract Oncol 2009 6 2 68 9 19092801 10.1038/ncponc1276

[B28] Catriona H Jamieson Y Chronic myeloid leukemia stem cell Hematology Am Soc Hematol Educ Program 2008 34 436 42 10.1182/asheducation-2008.1.436 19074122

[B29] Pelletier SD Hong DS Hu Y Liu Y Li S Lack of the adhesion molecules P-selectin and intercellular adhesion molecule-1 accelerate the development of BCR/ABL-induced chronic myeloid leukemia-like myeloproliferative disease in mice Blood 2004 104 2163 2171 10.1182/blood-2003-09-3033 15213099

[B30] Martin-Henao GA Quiroga R Sureda A González JR Moreno V García J L-selectin expression is low on CD34+ cells from patients with chronic myeloid leukemia and interferon-a up-regulates this expression Haematologica 2000 85 139 146 10681720

[B31] Wertheim JA Forsythe K Druker BJ Hammer D Boettiger D Pear WS BCR-ABL-induced adhesion defects are tyrosine kinase-independent Blood 2002 99 11 4122 4130 10.1182/blood.V99.11.4122 12010816

[B32] Fiore Emilio Fusco Carlo Romero Pedro Matrix metalloproteinase 9 (MMP-/gelatinase B) proteolytically cleaves ICAM-1 and participates in tumor cell resistance to natural killer cell-mediated cytotoxicity Oncogene 2002 21 5213 5223 10.1038/sj.onc.1205684 12149643

[B33] Darai E Stefanidakis M Koivunen E Cell-surface association between matrix metalloproteinases and integrins: role of the complexes in leukocyte migration and cancer progression Blood 2006 108 1441 1450 10.1182/blood-2006-02-005363 16609063

[B34] Molica S Vitelli G Levato D Giannarelli D Vacca A Cuneo A Cavazzini F Squillace R Mirabelli R Digiesi G Increased serum levels of matrix metalloproteinase-9 predict clinical utcome of patients with early B-cell chronic lymphocytic leukemia European Journal of Haematology 2003 10 373 378 10.1034/j.1600-0609.2003.00064.x 12756019

[B35] Kamiguti AS Lee ES Till KJ Harris RJ Glenn MA Lin K Chen HJ Zuzel M Cawley JC The role of matrix metalloproteinase 9 in the pathogenesis of chronic lymphocytic leukaemia Br J Haematol 2004 125 128 140 10.1111/j.1365-2141.2004.04877.x 15059134

[B36] Møller GM Frost V Melo JV Chantry A Upregulation of the TGFbeta signalling pathway by Bcr-Abl: implications for haemopoietic cell growth and chronic myeloid leukaemia FEBS Lett 2007 581 7 1329 34 10.1016/j.febslet.2007.02.048 17349636

[B37] Atfi A Abécassis L Bourgeade MF Bcr-Abl activates the AKT/Fox O3 signalling pathway to restrict transforming growth factor-beta-mediated cytostatic signals EMBO Rep 2005 6 10 985 91 10.1038/sj.embor.7400501 16113647 PMC1369182

[B38] Naka K Hoshii T Muraguchi T Tadokoro Y Ooshio T Kondo Y Nakao S Motoyama N Hirao A TGF-beta-FOXO signalling maintains leukaemia-initiating cells in chronic myeloid leukaemia Nature 2010 463 7281 676 80 10.1038/nature08734 20130650

[B39] Zhao ZG Li WM Chen ZC You Y Zou P Immunosuppressive properties of mesenchymal stem cells derived from bone marrow of patients with chronic myeloid leukemia Immunol Invest 2008 37 7 726 39 10.1080/08820130802349940 18821219

